# Joint effect of overweight/obesity and tobacco exposure on hypertension in children aged 6–17 years: a cross-sectional study

**DOI:** 10.3389/fped.2023.1188417

**Published:** 2023-06-30

**Authors:** Huan Gu, Long Hao, Mingxi Li, Ji Li

**Affiliations:** ^1^ Department of Integrative Cardiology, China-Japan Friendship Hospital, Beijing, China; ^2^ Department of Pediatrics, Beijing Fangshan District Liangxiang Hospital, Beijing, China; ^3^ Department of Pediatrics, Guang’ Anmen Hospital, China Academy of Chinese Medical Sciences, Beijing, China

**Keywords:** children, overweight/ obesity, tobacco exposure, hypertension, joint effect

## Abstract

**Aim:**

To assess the individual effects of overweight/obesity and tobacco exposure, and their combined effects on hypertension in children.

**Methods:**

This cross-sectional study included 6,339 children aged 6–17 years from National Health and Nutrition Examination Surveys 1999–2018. Participants’ height, weight and blood pressure (BP) were measured by trained technicians. Hypertension was defined as: mean systolic BP (SBP) and/or diastolic BP (DBP) ≥ 90th percentile for sex, age, and height (for children aged 1–13 years), and SBP ≥120 mmHg and/or a DBP ≥80 mmHg (for adolescents aged 13–17 years); or self-reported having been diagnosed with hypertension or taking antihypertensive medication. Gender- and age-specific body mass index (BMI) cut-points were used to define overweight/obesity: “overweight” was defined as a BMI > 1 standard deviation (SD); “obesity” was defined as BMI > 2SD; and “thinness” was defined as BMI < −2SD. Tobacco exposure was defined as having serum cotinine levels >0.05 µg/L or reporting the presence of at least one smoker in the household. Weighted univariate and multivariate logistic regression models were used to assess overweight/obesity and tobacco exposure with the odds of hypertension, and the combined effects of overweight/ obesity and tobacco exposure on hypertension, followed by strata-specific analyses. Odds ratios (OR) with 95% confidence intervals (CI) were calculated.

**Results:**

The prevalence of overweight/obesity and tobacco exposure was significantly higher in the hypertension group than in the non-hypertension group. Overweight/obesity (*OR* = 1.67, 95%CI: 1.26–2.21/ *OR* = 2.38, 95%CI: 1.67–3.39) and tobacco exposure (*OR* = 1.58, 95%CI: 1.16–2.14) were associated with a higher odd of hypertension in children, respectively. Additionally, we also observed the combined effect between overweight (*OR* = 3.05, 95%CI: 1.96–4.75)/obesity (*OR* = 3.68, 95%CI: 2.24–6.03) and tobacco exposure were related to hypertension odds in children, with a significant effect in different populations.

**Conclusion:**

There may exist joint effect of overweight/obesity and tobacco exposure on the odds of hypertension in American children. These findings offer an insight that early weight control and reduction of tobacco exposure may be important to reduce odds of hypertension in children.

## Introduction

Hypertension in children has become a major global public health problem (1). A large amount of research evidence shows that childhood hypertension is closely related to hypertension and cardiovascular disease in adults (2, 3). Therefore, it is important to prevent and manage the development of hypertension in children.

The occurrence of hypertension in children is influenced by many factors. Previous research has shown that overweight and obese children were more likely to develop hypertension than underweight or normal-weight children (4, 5). A meta-analysis also concluded that childhood overweight and obesity were associated with elevated blood pressure (BP), and controlling childhood obesity is beneficial in lowering BP (6). Tobacco exposure dramatically increases BP by activating the sympathetic nervous system (7). Recent evidence suggests that tobacco exposure plays an important role in the development of hypertension (8, 9). In the study of Levy RV, et al, they found that tobacco exposure was associated with both elevated BP and hypertension in children and adolescents (10). Liu SH, et al., also reported that higher diastolic blood pressure (DBP) in association with secondhand tobacco smoke exposure during childhood (11).

Interestingly, several studies have found a combined effect of body fat/overweight and tobacco exposure on hypertension in adults. A cross-sectional study showed that there was an interaction between lipid accumulation product (LAP) and smoking on hypertension risk, and the risk of hypertension in male with higher LAP and smoking was 3.32 times that of men with lower LAP and non-smoking (12). Shen et al. observed that the synergistic effect of secondhand smoke exposure and vitamin D deficiency on hypertension was influenced by body mass index (BMI), and the synergistic effect was particularly pronounced in overweight women (13). This interactive effect may be due to the shared effects of obesity and tobacco exposure on airway or systemic oxidative stress and inflammation (12, 13). However, there was few studies to explore the joint effect of overweight/obesity and tobacco exposure on the odds of hypertension risk in children and adolescents.

Here, this study intends to explore the combined effects of overweight/ obesity and tobacco exposure on children’s hypertension, which provided a scientific basis for the prevention and management of hypertension in children.

## Participants and methods

### Study participants

In this cross-sectional study, we analyzed publicly available data from the National Health and Nutrition Examination Survey (*N*HANES) between 1999 and 2018. NHANES is a program of studies conducted by the National Center for Health Statistics (*N*CHS). The survey combines interviews (demographic, socioeconomic, dietary, and health-related questions) and physical examinations (medical, dental, physiological measurements and laboratory tests) (13). Since our study was a secondary analysis for NHANES public database, ethical approval from Guang’ anmen Hospital, China Academy of Chinese Medical Sciences was not required for this study. Informed consent to participate in this study was provided by the participants’ legal guardian/next of kin.

**Figure 1:**
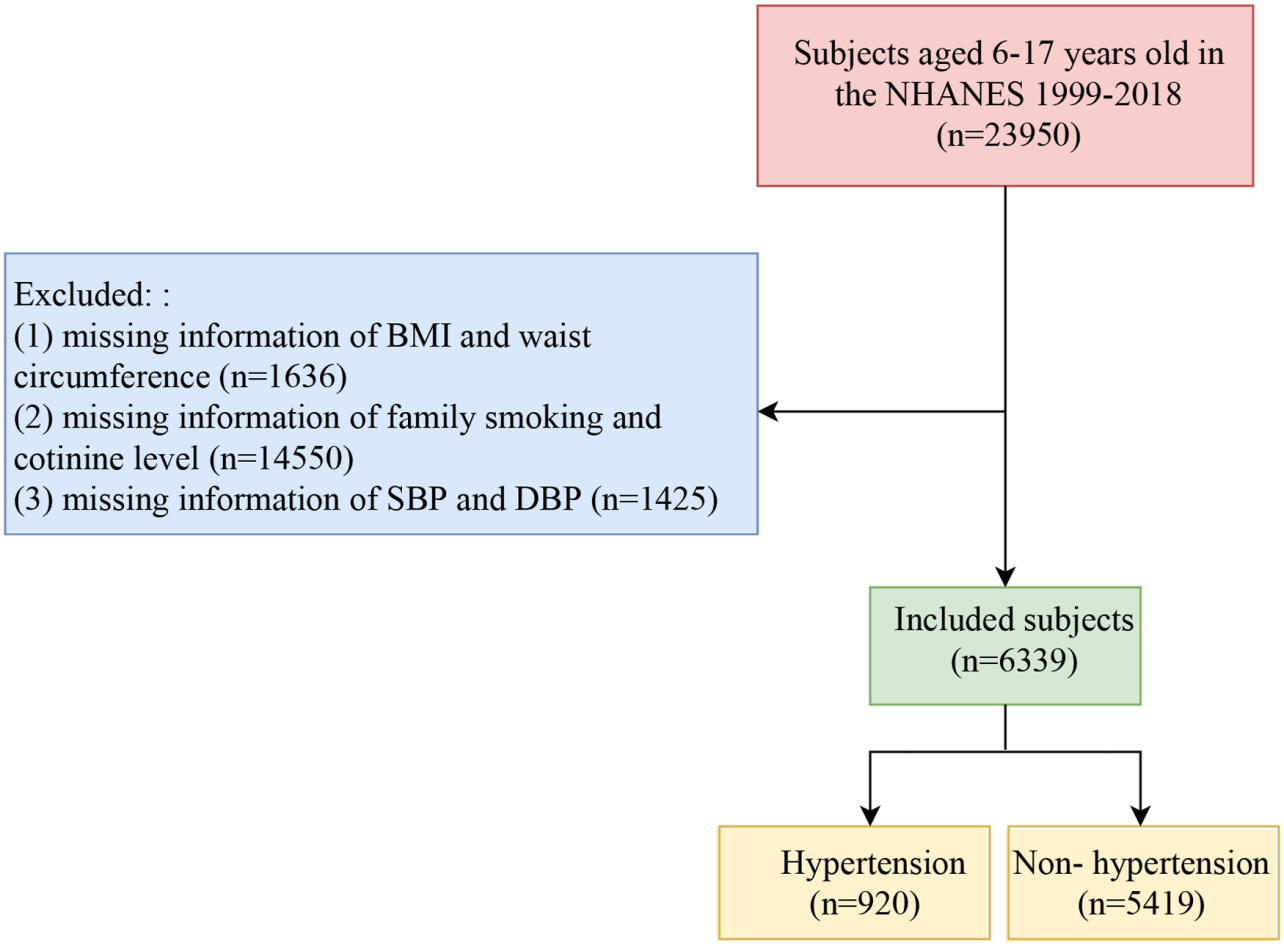
The flow chart of population selection.

In the current analysis, the inclusion criterion was subjects aged 6–17 years. The exclusion criteria were as follows: (1) subjects with missing information of BMI and waist circumference (*n* = 1,636); (2) subjects with missing information of family smoking and cotinine level (*n* = 14,550); (3) subjects with missing information of systolic blood pressure (SBP) and diastolic blood pressure (DBP) (*n* = 1,425). Finally, a total of 6,339 subjects were included in this study. The flow chart of population selection was shown in Figure 1.

### Definition of hypertension

For participants in the NHANES database, three consecutive BP measurements (SBP and DBP; Baumanometer® calibrated mercury true gravity wall model sphygmomanometer) were taken after a trained BP examiner asked the participants to sit quietly and rest for five minutes (14). For participants with three measurements, the BP record is the average of the last two measurements; for those with two or one measurement, the BP record is based on the last measurements. All BP measurements (systolic and diastolic) were conducted in a Mobile Examination Center (MEC). Hypertension is defined as: (1) mean SBP and/or DBP ≥90th percentile for sex, age, and height (for children aged 1–13 years), and SBP ≥120 mmHg and/or a DBP ≥80 mmHg (for adolescents aged 13–17 years) according to the 2017 American Academy of Pediatrics guidelines (15). (2) self-reported having been diagnosed with hypertension or taking antihypertensive medication. Either of these two conditions can be diagnosed as hypertension.

### Assessment of overweight/obesity and tobacco exposure

In the NHANES database, measurements of participants’ height (Stadiometer) and weight (Digital weight scale) were collected by trained health technicians in the MEC. Participants of all ages reported their weight in kilograms, while those aged 2 and older recorded their height in centimeters (https://wwwn.cdc.gov/Nchs/Nhanes/2009−2010/BMX_F.htm). BMI are calculated for NHANES participants follows: weight (kg) divided by the square of height (m). According to the World Health Organization (WHO 2007 Z-score) and the International Obesity Task Force (IOTF2000), gender- and age-specific BMI cut-points were used to define overweight/obesity (16): “overweight” was defined as a BMI > 1 standard deviation (SD); “obesity” was defined as BMI > 2SD; and “thinness” was defined as BMI < −2SD.

Cotinine serves as the primary metabolite of nicotine. In this study, we employed both objective and self-reported measures to evaluate tobacco exposure (17). Subjects were categorized as tobacco exposure if their serum cotinine levels were >0.05 µg/L or if they reported the presence of at least one smoker in their household; subjects were classified as non-tobacco exposure if their serum cotinine levels were ≤0.05 µg/L and no smokers were reported in the household.

### Data collection

Some variables of the participants were extracted: age (years), gender, race, education level, BMI (kg/m^2^), waist circumference (cm), income, screen time (h/day), energy intake, maternal smoking during pregnancy, birth weight (pounds), childhood smoking (active smoking, passive smoking), cotinine (µg/l). Screen time was considered as the time spent watching TV/videos and playing computer games (18); and the screen time was categorized as high-level group (≥3 h/day) and low-level group (<3 h/day).

### Statistical analysis

In this study, Continuous variables with normal distribution were described as weighted mean (standard error) [Mean (SE)], and independent samples t-test for comparison between two groups. Categorical variables were described as weighted percentages and frequencies [n (%)], and the chi-square test was used for the comparison between groups; rank data required the use of rank sum test. Statistical analyses were performed using SAS 9.4 software (SAS Institute Inc., Cary, NC, USA), R 4.2.2 software and Python 3.9.12 software. *P *< 0.05 was considered statistically significant.

We used the weighted univariate and multivariate logistic regression models to examine the association of overweight/obesity and tobacco exposure with the odds of hypertension in children, respectively. The multivariate logistic regression model adjusted for gender, education level, waist circumference, maternal smoking during pregnancy and birth weight. Importantly, we assessed the combined effects of overweight/ obesity and tobacco exposure on hypertension in children. Odds ratios (OR) with 95% confidence intervals (CI) were calculated. Simultaneously, this joint effect has also been investigated in different populations. In this study, random forest interpolation method was used to fill the missing variables, and the difference analysis was conducted on the data before and after interpolation (Supplementary Table S1).

## Results

### Basic characteristics

Table 1 presents the basic characteristics of all eligible participants (*n* = 6,339). The mean age was 12.68 years old, and 51.66% were males. In this population, the prevalence of hypertension was approximately 14.51%. We compared the differences in characteristics between the hypertension group and non-hypertension group. Individuals with hypertension had a higher rate of overweight/obesity than those without hypertension (24.07%/40.83 vs. 22.36%/20.24, *P *< 0.001), as well as the rate of maternal smoking during pregnancy (20.78% vs. 19.28%, *P *< 0.001). Compared with participants without hypertension, those with hypertension tended to the tobacco exposure (*P* < 0.001). Additionally, we found significant differences between the two groups for some variables, including gender, education level, waist circumference, maternal smoking during pregnancy and birth weight, which were also considered as confounding factors in this study.

**Table 1:** The basic characteristics of participants.

Variables	Total (*n* = 6339)	Non-Hypertension (*n* = 5419)	Hypertension (*n* = 920)	*P*
Age, years, Mean (S.E)	12.68 (0.05)	12.64 (0.05)	12.94 (0.14)	0.051
Gender, *n* (%)				<0.001
Male	3193 (51.66)	2613 (49.71)	580 (64.08)	
Female	3146 (48.34)	2806 (50.29)	340 (35.92)	
Race, *n* (%)				0.060
Mexican American	1241 (12.96)	1050 (12.74)	191 (14.36)	
Non-Hispanic Black	1903 (15.19)	1598 (14.69)	305 (18.35)	
Non-Hispanic White	1892 (56.56)	1637 (57.21)	255 (52.44)	
Other Hispanic	530 (6.85)	461 (6.92)	69 (6.44)	
Other Race—Including Multi-Racial	773 (8.44)	673 (8.44)	100 (8.41)	
Education level, *n* (%)				<0.001
Less than 6th grade	3454 (51.51)	3002 (51.96)	452 (48.69)	
7th to 9th grade	2019 (33.22)	1732 (33.68)	287 (30.31)	
9th to 12th grade	840 (14.83)	668 (14.03)	172 (19.97)	
High school and above	26 (0.43)	17 (0.33)	9 (1.04)	
BMI, kg/m^2^, Mean (S.E)	22.13 (0.11)	21.68 (0.10)	24.97 (0.33)	<0.001
Waist circumference, cm, Mean (S.E)	76.60 (0.30)	75.51 (0.29)	83.51 (0.80)	<0.001
Income, *n* (%)				0.125
Over $20,000	4513 (78.20)	3877 (78.66)	636 (75.30)	
Under $20,000	1642 (19.49)	1383 (19.00)	259 (22.57)	
Unknown	184 (2.31)	159 (2.34)	25 (2.13)	
Screen time, *n* (%)				0.781
High-level	3412 (51.90)	2915 (51.81)	497 (52.47)	
Low-level	2927 (48.10)	2504 (48.19)	423 (47.53)	
Energy intake, *n* (%)				0.300
High-level	1475 (23.74)	1293 (24.13)	182 (21.27)	
Low-level	3603 (59.64)	3065 (59.48)	538 (60.66)	
Unknown	1261 (16.62)	1061 (16.39)	200 (18.07)	
Maternal smoking during pregnancy, *n* (%)				<0.001
No	3985 (60.02)	3474 (61.44)	511 (50.96)	
Yes	1134 (19.48)	976 (19.28)	158 (20.78)	
Unknown	1220 (20.50)	969 (19.28)	251 (28.25)	
Childhood smoking, *n* (%)				0.165
Active smoking	383 (6.56)	315 (6.28)	68 (8.30)	
Passive smoking	5956 (93.44)	5104 (93.72)	852 (91.70)	
Birth weight, pounds, *n* (%)				<0.001
<5.5	743 (10.29)	629 (9.95)	114 (12.44)	
5.5−8.9	3850 (60.44)	3354 (61.50)	496 (53.72)	
>8.9	397 (6.59)	352 (6.99)	45 (4.08)	
Unknown	1349 (22.68)	1084 (21.56)	265 (29.76)	
Cotinine, µg/L, *n* (%)				<0.001
≤0.05	2622 (46.40)	2304 (47.87)	318 (37.00)	
>0.05	3717 (53.60)	3115 (52.13)	602 (63.00)	
Tobacco exposure, *n* (%)				<0.001
No	2246 (40.36)	1980 (41.83)	266 (31.06)	
Yes	4093 (59.64)	3439 (58.17)	654 (68.94)	
BMI, *n* (%)				<0.001
Obesity	1559 (23.04)	1168 (20.24)	391 (40.83)	
Overweight	1456 (22.59)	1249 (22.36)	207 (24.07)	
Thinness/Normal	3324 (54.37)	3002 (57.40)	322 (35.10)	

BMI, body mass index; SE, standard error.

Tobacco exposure: serum cotinine levels were >0.05 µg/L or reported the presence of at least one smoker in their household (Yes); serum cotinine levels were ≤0.05 µg/L and no smokers were reported in the household (No).

### The association of overweight/obesity and tobacco exposure with the risk of hypertension in children

As shown in Table 2, we assessed the association of overweight/obesity and tobacco exposure on hypertension, respectively. In the weighted univariate logistic regression model, compared with thinness/normal group, the OR for the relationship of overweight and hypertension odds was 1.76 (95%CI: 1.40−2.21); and the OR for the relationship of obesity and hypertension odds was 3.30 (95%CI: 2.64−4.12). After adjusting confounding factors, overweight (*OR* = 1.67, 95%CI: 1.26−2.21, *P *= 0.001) and obesity (*OR* = 2.38, 95%CI: 1.67−3.39, *P *< 0.001) remained associated with odds of hypertension in children. Similarly, tobacco exposure has been found to be related to the odds of hypertension in children in the weighted multivariate logistic regression model (*OR* = 1.58, 95%CI: 1.16−2.14, *P *= 0.004).

**Table 2:** The association of overweight/obesity and tobacco exposure with the risk of hypertension.

Variables	Univariate model^a^		Multivariate model^b^	
	OR (95%CI)	*P*	OR (95%CI)	*P*
BMI				
Thinness/Normal	Ref		Ref	
Overweight	1.76 (1.40–2.21)	<0.001	1.67 (1.26–2.21)	0.001
Obesity	3.3 (2.64–4.12)	<0.001	2.38 (1.67–3.39)	<0.001
Tobacco exposure				
No	Ref		Ref	
Yes	1.6 (1.25–2.03)	<0.001	1.58 (1.16–2.14)	0.004

BMI, body mass index; Ref, reference, OR, odds ratio, CI, confidence interval.

^a^ no adjustment for covariates;.

^b^ adjusted for gender, education level, waist circumference, maternal smoking during pregnancy and birth weight.

### Combined effects analysis

We divided the entire population into six groups based on gender- and age-specific BMI level and whether tobacco exposure was present (group 1: thinness/normal and non-tobacco exposure; group 2: overweight and non-tobacco exposure; group 3: obesity and non-tobacco exposure; group 4: thinness/normal and tobacco exposure; group 5: overweight and tobacco exposure; group 6: obesity and tobacco exposure). As expressed in Table 3, using thinness/normal and non-tobacco exposure (group 1) as the reference, the OR for the relationship of the other five groups and hypertension risk was 1.96 (95%CI: 1.26−3.03, *P *= 0.003, group 2), 4.71 (95%CI: 3.34−6.63, *P *< 0.001, group 3), 1.93 (95%CI: 1.36−2.74, *P *< 0.001, group 4), 3.24 (95%CI: 2.32, *P *< 0.001, group 5) and 5.20 (95%CI: 3.68−7.35, *P *< 0.001, group 6), respectively. After adjusting confounding factors, “obesity and non-tobacco exposure” (*OR* = 3.08, 95%CI: 2.03−4.67, *P *< 0.001, group 3), “thinness/normal and tobacco exposure” (*OR* = 1.76, 95%CI: 1.14−2.72, *P *= 0.012, group 4), “overweight and tobacco exposure” (*OR* = 3.05, 95%CI: 1.96−4.75, *P *< 0.001, group 5) and “obesity and tobacco exposure” (*OR* = 3.68, 95%CI: 2.24−6.03, *P *< 0.001, group 6) were still associated with the odds of hypertension in children. The result also indicated that the combined effects of overweight/obesity and tobacco exposure were associated with the odds of hypertension among children.

**Table 3:** Combined effects analysis.

Interaction term	Univariate logistic regression model^a^		Multivariate logistic regression model^b^	
	OR (95%CI)	*P*	OR (95%CI)	*P*
BMI*Tobacco exposure				
Thinness/Normal *No	Ref		Ref	
Overweight^a^No	1.96 (1.26–3.03)	0.003	1.57 (0.94–2.63)	0.088
Obesity^a^ No	4.71 (3.34–6.63)	<0.001	3.08 (2.03–4.67)	<0.001
Thinness/Normal^a^Yes	1.93 (1.36–2.74)	<0.001	1.76 (1.14–2.72)	0.012
Overweight^a^Yes	3.24 (2.32–4.51)	<0.001	3.05 (1.96–4.75)	<0.001
Obesity^a^Yes	5.2 (3.68–7.35)	<0.001	3.68 (2.24–6.03)	<0.001

BMI, body mass index; Ref, reference; OR, odds ratio; CI, confidence interval.

^a^ no adjustment for covariates.

^b^ adjusted for gender, education level, waist circumference, maternal smoking during pregnancy and birth weight.

### Subgroup analysis based on age and gender

**Figure 2:**
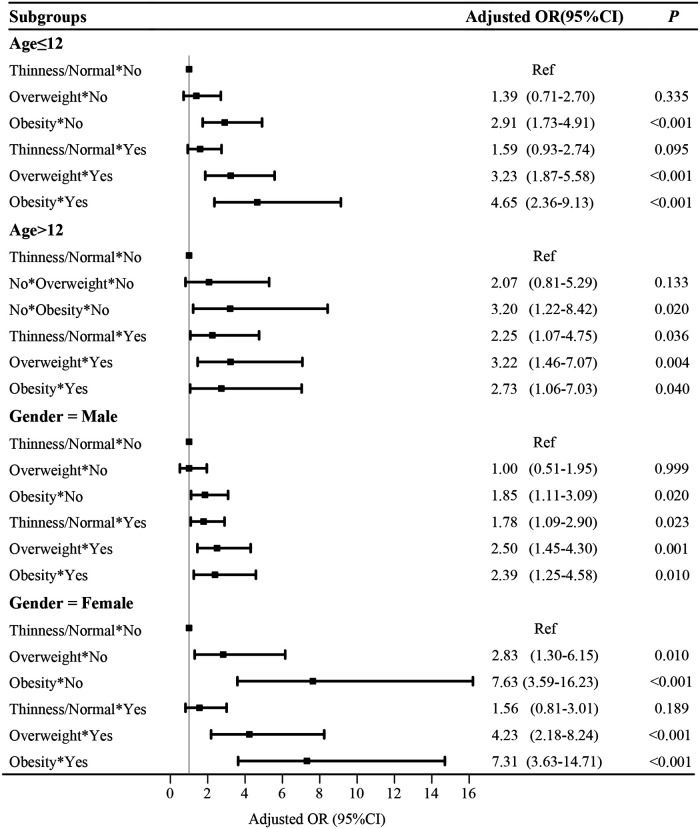
Subgroup analysis based on age and gender.

After adjusting confounding factors, the combined effects of overweight/ obesity and tobacco exposure with the odds of hypertension were assessed in different populations (Figure 2). In the age ≤12 years group, “obesity and non-tobacco exposure”, “overweight and tobacco exposure” and “obesity and tobacco exposure” were associated with the odds of hypertension in children. In the age >12 years group, “obesity and non-tobacco exposure”, “thinness/normal and tobacco exposure”, “overweight and tobacco exposure” and “obesity and tobacco exposure” were associated with the odds of hypertension in children. In the stratification by gender, we also observed the combined effects of overweight/obesity and tobacco exposure on the odds of hypertension in children.

## Discussion

Our study suggested the independent relationship between overweight/obesity, tobacco exposure and the odds of children’s hypertension. More importantly, this study indicated the joint effect between overweight/obesity and tobacco exposure towards the odds of hypertension in children. These findings offer a new insight that early weight control and reduction of tobacco exposure may be important to reduce the odds of hypertension in children.

In this study, the prevalence of overweight, obesity and hypertension were approximately 22.59%, 23.04% and 14.51%, respectively. Studies conducted in China reported that the prevalence rates of childhood overweight and obesity were 10.90% and 8.70%, respectively (19); while the prevalence rate of hypertension was approximately 13.56% (20). The difference in prevalence may be related to variations in dietary habits across different countries. Our study is one of those supporting the evidence that overweight/ obesity was related to the odds of hypertension in children. Several mechanisms might be used to explain this relationship: For overweight/ obesity populations, adipocytes produce a hormone called leptin, which can bind to leptin receptors in the central nervous system to increase sympathetic nerve activity, leading to hypertension development (21, 22). Obesity can lead to increased sympathetic nervous system activity (23). In addition, dietary factors, endothelial and vascular dysfunction, neuroendocrine imbalances, metabolic, sodium retention, and inflammation have all been identified as potential mechanisms for the association of obesity with hypertension (24–26). Several researchers have proposed a relationship between tobacco exposure and the odds of hypertension (10, 27, 28). The result of our study was consistent with previous studies. Skipina TM, et al. also pointed out that cigarette smoke is able to induce cardiovascular mitochondrial oxidative stress, promote endothelial dysfunction, and contribute to the development of hypertension (8). In this study, we found that there was a higher rate of tobacco exposure in the hypertension group than non-hypertension group. After adjusting confounding factors, tobacco exposure was considered to be related to the odds of hypertension in children. It is worth mentioning that, “thinness/normal and tobacco exposure” was also found to be associated with the odds of hypertension in children, which also means that tobacco exposure has an effect on hypertension in children regardless of BMI level.

A recent cross-sectional study reported that there was an interactive effect between secondhand smoke exposure and overweight on the prevalence of hypertension among non-smoking Chinese coke oven workers and NHANES participants (29). To our knowledge, there has been no clear conclusion about the overweight/obesity and tobacco exposure on hypertension odds in children and adolescents so far. In the present study, we observed the joint effect between overweight/obesity and tobacco exposure with hypertension odds in children. Potential mechanisms may involve the effects of airway or systemic oxidative stress and inflammation (12, 13, 29). Not only that, the results of subgroup analysis based on age and gender also indicated that the joint effect of thinness/normal and tobacco exposure on the risk of hypertension was not significant among children younger than 12 years old and female children. The reason may be related to the lower level of tobacco exposure in this population.

Nevertheless, this study had certain limitations. Firstly, this was a cross-sectional study, we were unable to assess the causal relationship between overweight/ obesity, tobacco exposure and hypertension in children. Secondly, although we controlled for some potential confounders, some possible confounders that were not included in the NHANES database, such as parental hypertension and obesity status, remained. Lastly, the study had a limited sample size of active smoking children, and the gender and age of active smokers were not included in the analysis. Further prospective studies are needed to confirm these findings.

## Conclusion

In summary, overweight/obesity and tobacco exposure were associated with higher odds of hypertension in children, respectively. There exist joint effect of overweight/obesity and tobacco exposure on the odds of hypertension in American children. These findings offer a new insight that early weight control and reduction of tobacco exposure may be important to reduce odds of hypertension in children.

## Data Availability

Publicly available datasets were analyzed in this study. This data can be found here: NHANES database, https://wwwn.cdc.gov/nchs/nhanes/.
