# Supplementary material for: Joint effect of overweight/obesity and tobacco exposure on hypertension in children aged 6–17 years: a cross-sectional study
Source: Front Pediatr. 2023 Jun 30;11:1188417. doi: 10.3389/fped.2023.1188417 (PMC10347524; doi:10.3389/fped.2023.1188417)
Supplement: Supplementary file 1 [file Table1.docx]

**Supplementary Table 1 The difference analysis for before and after data interpolation**

| **Variables** | **Before interpolation (n=6339)** | **After interpolation (n=6339)** | ***P*** |
| --- | --- | --- | --- |
| Education, n (%) |  |  | 0.672 |
| Less than 6th grade | 3453 (51.52) | 3454 (51.51) |  |
| 7th to 9th grade | 2016 (33.21) | 2019 (33.22) |  |
| 9th to 12th grade | 840 (14.84) | 840 (14.83) |  |
| High school and above | 26 (0.43) | 26 (0.43) |  |
